# Risk of macrosomia remains glucose-dependent in a cohort of women with pregestational type 1 diabetes and good glycemic control

**DOI:** 10.1007/s12020-016-1134-z

**Published:** 2016-10-11

**Authors:** Katarzyna Cyganek, Jan Skupien, Barbara Katra, Alicja Hebda-Szydlo, Izabela Janas, Iwona Trznadel-Morawska, Przemysław Witek, Elżbieta Kozek, Maciej T. Malecki

**Affiliations:** 1 Department of Metabolic Diseases, University Hospital, Krakow, Poland; 2 Department of Metabolic Diseases, Jagiellonian University Medical College, Krakow, Poland

**Keywords:** Type 1 diabetes, Pregnancy, Macrosomia, Glycated hemoglobin A_1c_

## Abstract

Macrosomia risk remains high in type 1 diabetes (T1DM) complicated pregnancies. A linear relationship between macrosomia risk and glycated hemoglobin A_1c_ (HbA_1c_) was described; however, low range of HbA_1c_ has not been studied. We aimed to identify risk factors and examine the impact of HbA_1c_ on the occurrence of macrosomia in newborns of T1DM women from a cohort with good glycemic control. In this observational retrospective one-center study we analyzed records of 510 consecutive T1DM pregnancies (1998–2012). The analyzed group consisted of 375 term singleton pregnancies. We used multiple regression models to examine the impact of HbA_1c_ and self-monitored glucose in each trimester on the risk of macrosomia and birth weight. The median age of T1DM women was 28 years, median T1DM duration—11 years, median pregestational BMI—23.3 kg/m^2^. Median birth weight reached 3520 g (1st and 3rd quartiles 3150 and 3960, respectively) at median 39 weeks of gestation. There were 85 (22.7 %) macrosomic (>4000 g) newborns. Median HbA_1c_ levels in the 1st, 2nd, and 3rd trimester were 6.4, 5.7, and 5.6 %. Third trimester HbA_1c_, mean fasting self-monitored glucose and maternal age were independent predictors of birth weight and macrosomia. There was a linear relationship between 3rd trimester HbA_1c_ and macrosomia risk in HbA_1c_ range from 4.5 to 7.0 %. Macrosomia in children of T1DM mothers was common despite excellent metabolic control. Glycemia during the 3rd trimester was predominantly responsible for this condition.

## Introduction

Diabetes is the most common metabolic disease complicating pregnancy [1, 2]. It is associated with increased risk of maternal and neonatal complications and constitutes a serious medical, social, and financial problem. There is ample evidence that regardless of diabetes type, hyperglycemia during pregnancy increases risk of adverse outcomes [3, 4]. Today pregnant women with type 1 diabetes (T1DM) can achieve a very good glycemic control, however, despite substantial improvements in diabetes care, the rate of complications, and particularly macrosomia, remains high [5–8]. Fetal macrosomia is associated with an increased risk of cesarean section, injuries to the birth canal and to the fetus, as well as obesity and metabolic diseases later in life [9–13].

The HAPO study (Hyperglycemia and Adverse Pregnancy Outcomes) revealed that there was no risk threshold in the association of fetal macrosomia and glycemia measured during an oral glucose tolerance test performed in pregnant women without pregestational diabetes [14]. This observation, together with another clinical trial of glucose lowering treatment in mild gestational diabetes [15], have led to conclusion that therapeutic measures to decrease blood glucose in pregnancy should be addressed to a larger population of pregnant women. It subsequently resulted in a substantial change of diagnostic criteria of gestational diabetes worldwide. In pregestational diabetes the target hemoglobin A_1c_ (HbA_1c_) during pregnancy is generally defined at a close to normal level, for example <6.5 % (<47 mmol/mol) according to The National Institute for Health and Care Excellence [16] or within the range of 6.0–6.5 % (42–47 mmol/mol) according to the current standards of diabetes care of The American Diabetes Association [17]. In the light of the HAPO study results, one can hypothesize that there is an analogous continuous relationship between birth weight and HbA_1c_ level in women with T1DM. This association could be present for HbA_1c_ values below 6 % (42 mmol/mol), prompting reconsideration of current therapeutic targets.

We have earlier reported that women with T1DM achieved excellent glycemic control in the 2nd and 3rd trimesters of pregnancy, regardless their insulin treatment regimen—multiple daily injections or personal insulin pump [18]. In the current observational study we describe the relationship between maternal HbA_1c_, self-monitored glucose and macrosomia risk in this group, and attempt to verify the hypothesis that macrosomia remains a glucose-dependent complication of pregnancy in women with T1DM, in spite of achievement of therapeutic goals.

## Subjects and methods

### Subjects

This retrospective, observational, cross-sectional cohort study was performed at the Department of Metabolic Diseases of the University Hospital, Krakow, Poland, an academic referral center for diabetes care in South-Eastern Poland. All pregnant women with pre-existing T1DM were registered between the years 1998 and 2012 as a continuous case series. They were all referred to the Department no later than in the 1st trimester of pregnancy and had a clinical diagnosis of T1DM established at least one year prior to conception. The patients were Caucasian residents of South-Eastern Poland.

The study protocol and its conduct were concordant with the Helsinki Declaration and it was approved and supervised by the Jagiellonian University Bioethical Committee.

All women with diabetes who were pregnant or planned pregnancy received intensive diabetes management care in the clinic as described earlier [18]. That management involved education on diet and physical activity, glycemic goals and self-monitoring of blood glucose (SMBG), with self-adjustment of insulin dose. According to the Polish Diabetes Association recommendations at the time when the study was performed [19], the therapeutic targets for all women were: (a) HbA1c < 6.1 % (43 mmol/mol), (b) fasting self-monitored glucose 60–90 mg/dl, and (c) subsequent pre- and 1-h postprandial self-monitored within 60–120 mg/dl. Pregnancy planning was defined as described earlier [18]. Briefly, the term “pregnancy planning” refers to the time of entry to our intensive diabetes management program and applies to women who entered this program before conception.

Clinical characteristics of the women, information on the course of pregnancy and glycemic control markers were collected during regular clinic visits. Diabetic retinopathy was diagnosed by funduscopy at a visit scheduled during the 1st trimester and diabetic nephropathy was diagnosed with urinary albumin and serum creatinine (eGFR) according to KDIGO guidelines [20]. Pregnancy outcomes were ascertained during a postpartum visit and from medical records. Birth weight was recorded for all term pregnancies. Macrosomia was defined as birth weight >4000 g.

HbA_1c_ was measured with high performance liquid chromatography (HLPC) on a Variant apparatus (Biorad) and was DCCT adjusted. The inter- and intra-assay coefficient of variation was less than 2 %. The clinical laboratory in which HbA_1c_ was measured participates in an internal and external quality control program by regularly performing calibration procedures and blind assays of standardized material.

In each trimester the patient-provided SMBG profile was recorded during a study visit—it included measurements from 7 days preceding the visit. The measurements recorded were typically at least 8 times a day: before and 60 min after every meal, additionally at bedtime and between 2–4 am. Corresponding timed non-missing measurements from consecutive days were averaged. The following variables were extracted or calculated from SMBG profiles in each trimester: mean fasting glucose, mean glucose (arithmetic mean of all measurements), mean postprandial glucose (arithmetic mean of postprandial measurements) and mean glucose amplitude (average difference between the highest and the lowest daily glucose measurement).

### Statistical analyses

For descriptive purposes the data is presented as medians (1st, 3rd quartile) and mean ± standard deviation (SD). Statistical comparisons between macrosomia and normal birth weight groups were performed with the Wilcoxon test, chi-square test of Fisher exact test, where applicable.

To identify independent risk predictors of macrosomia we used multiple logistic regression analysis. The complete list of independent variables included: maternal age, T1DM duration, presence of diabetic retinopathy or nephropathy, maternal weight and BMI before conception, weight gain during pregnancy, HbA_1c_ recorded in each of three trimesters, mean fasting self-monitored glucose, mean daily self-monitored glucose, mean postprandial self-monitored glucose and mean SMBG amplitude, treatment with continuous subcutaneous insulin infusion (CSII) and pregnancy planning. We performed a supervised best subset selection of candidate variables by minimizing Bayesian Information Criterion. We controlled collinearity with variance inflation and monitored the confounding properties of predictors. In the final model we retained all nominally significant predictors. In addition, the predictors from the final logistic model were evaluated with multiple linear regression model for their association with birth weight as a continuous variable. As a sensitivity analysis we fitted multiple logistic and linear regression models to data restricted to women who in all three trimesters had HbA_1c_ < 6.5 % (48 mmol/mol), the current American Diabetes Association (ADA) and National Institute for Health and Clinical Excellence (NICE) target; a similar analysis was done for HbA_1c_ < 6.1 % (43 mmol/mol) during the entire pregnancy.

To dissect how risk of macrosomia depends on different levels of HbA_1c_ in the 3rd trimester we stratified the distribution of this marker and calculated risk of macrosomia in each stratum. First, for graphical assessment we divided the cohort into six arbitrary groups. An apparent risk increase above HbA1c of 5.6 % (38 mmol/mol) was tested with a logistic spline model. Then, to obtain a smooth plot of the relationship between HbA_1c_ and macrosomia risk we divided the HbA_1c_ distribution into 15 equal strata, each containing 25 women. For each stratum we calculated mean 3rd trimester HbA_1c_ and probability of macrosomia. We used a penalized low-rank thin-plate spline model [21] fitted to a scatter of 15 macrosomia probabilities by mean 3rd trimester HbA_1c_, with knots placed at a regular distance of a 0.3 percentage point between HbA_1c_ 4.5 and 7.5 % (26–59 mmol/mol, 11 knots). The posterior probabilities of model parameters were estimated through Gibbs sampling (an example of Markov chain Monte Carlo algorithm). We used non-informative priors and initial values of fixed-effect parameters were estimated using restricted maximum likelihood. We used 30,000 burn-in iterations, and 100,000 iterations for inference, of which we saved 10,000. Convergence was assessed by visual inspection of trace plots and density plots from 4 parallel chains, autocorrelation of time-series and Gelman–Rubin diagnostic.

The analysis was performed with SAS 9.3 software (Cary, NC, USA), OpenBUGS 3.2.3, R software 3.1.0 (The R Foundation for Statistical Computing, Vienna, Austria) using SemiPar and R2OpenBUGS packages. *P*-values <0.05 were considered significant.

## Results

**Fig. 1:**
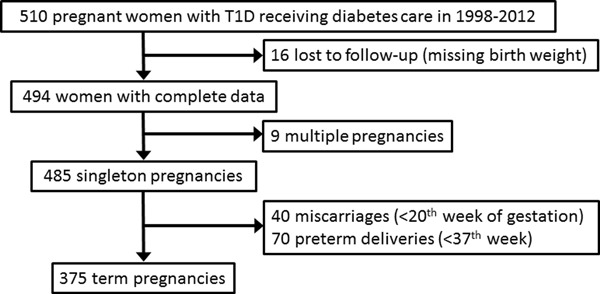
Numbers of patients initially accessed, excluded and included in the study

The exclusion criteria and causes of dropout from the initial sample size of 510 consecutive patients are presented in Fig. 1. Briefly, 16 women had incomplete follow-up and/or missing outcome data, 9 were excluded due to multiple pregnancy, in 110 women the pregnancy ended before the 37th week. The final analysis included 375 woman–child pairs. The baseline characteristics of the study population are presented in Table 1. The median age at first pregnancy visit was 28 years and T1DM duration was 11 years. The mothers had generally adequate body weight, with median BMI 23.3 kg/m^2^. Consistently with long T1DM duration, prevalence of retinopathy was 25.6 %. None of the patients showed evidence of chronic kidney disease of stage 3 or higher.

**Table 1:** Characteristics of the study group

Characteristics	Median (1st, 3rd quartile) and mean ± standard deviation or count (%)			*P*-value*
	No macrosomia *N* = 291	Macrosomia *N* = 84	Odds ratio (95 % CI)	
Maternal baseline characteristrics				
Age (years)	28 (25, 31)	27 (24, 30)	0.76 (0.58, 0.99)	0.026
	28 ± 4.6	27 ± 5.1	per 5 years ↑	
Diabetes duration (years)	11 (5, 18)	10 (5, 16)	0.86 (0.72, 1.02)	0.13
	12 ± 7.6	10 ± 6.5	per 5 years ↑	
BMI before conception (kg/m^2^)	23.2 (21.7, 25.6)	23.6 (22.0, 25,7)	1.04 (0.97, 1.11)	0.22
	23.9 ± 3.5	24.3 ± 3.4	per 1 kg/m^2^ ↑	
Retinopathy	82 (28.2 %)	14 (16.6 %)	0.51 (0.27, 0.95)	0.033
Nephropathy	5 (1.7 %)	2 (2.4 %)	1.39 (0.27, 7.32)	0.66
Treatment during pregnancy				
Pregnancy planning	122 (41.9 %)	34 (40.5 %)	1.07 (0.65, 1.78)	0.81
Analog insulin	174 (59.8 %)	52 (61.9 %)	1.08 (0.66, 1.79)	0.73
CSII	164 (56.4 %)	57 (67.9 %)	1.63 (0.98, 2.70)	0.059
Glycemic control in 1st trimester				
HbA1c (%) (mmol/mol)	6.3 (5.7, 7.2)	6.9 (6.0, 7,5)	1.13 (0.94, 1.35)	0.029
	6.6 ± 1.3	6.8 ± 1.1	per 1 % ↑	
	45 (39, 55)	52 (42, 59)		
	49 ± 15	51 ± 12		
Mean SMBG glucose (mmol/l)	5.9 (5.4, 6.4)	6.0 (5.5, 6.8)	1.06 (0.88, 1.26)	0.50
	6.1 ± 1.3	6.2 ± 1.3	per 1 mmol/l ↑	
Mean fasting glucose (mmol/l)	5.5 (4.6, 6.5)	6.1 (5.0, 7.2)	1.13 (1.00, 1.28)	0.012
	5.8 ± 1.8	6.3 ± 2.0	per 1 mmol/l ↑	
Glycemic control in the 2nd trimester				
HbA1c (%) (mmol/mol)	5.6 (5.1, 6.2)	6.0 (5.4, 6.5)	1.43 (1.12, 1.83)	0.002
	5.7 ± 0.9	6.1 ± 1.2	per 1 % ↑	
	38 (32, 44)	42 (36, 48)		
	39 ± 10	43 ± 13		
Mean SMBG glucose (mmol/l)	5.6 (5.2, 6.0)	6.0 (5.5, 6.4)	1.45 (1.14, 1.85)	<0.001
	5.7 ± 1.0	6.1 ± 1.0	per 1 mmol/l ↑	
Mean fasting glucose (mmol/l)	5.1 (4.5, 5.8)	5.7 (4.9, 6.4)	1.42 (1.18, 1.71)	<0.001
	5.3 ± 1.2	5.9 ± 1.6	per 1 mmol/l ↑	
Glycemic control in the 3rd trimester				
HbA1c (%) (mmol/mol)	5.5 (5.1, 6.0)	6.0 (5.4, 6.5)	1.81 (1.36, 2.41)	<0.001
	5.6 ± 0.8	6.1 ± 0.9	per 1 % ↑	
	37 (32, 42)	42 (37, 48)		
	38 ± 9	43 ± 10		
Mean SMBG glucose (mmol/l)	5.7 (5.1, 6.2)	5.9 (5.4, 6.5)	1.48 (1.15, 1.92)	0.001
	5.7 ± 0.9	6.1 ± 0.9	per 1 mmol/l ↑	
Mean fasting glucose (mmol/l)	5.0 (4.4, 5.7)	5.4 (4.9, 6.4)	1.52 (1.24, 1.87)	<0.001
	5.1 ± 1.1	5.8 ± 1.5	per 1 mmol/l ↑	
Follow-up outcomes				
Duration of pregnancy (weeks)	39 (38, 40)	39 (39, 41)	1.22 (1.03, 1.43)	0.029
	39 ± 1.4	40 ± 1.6	per 1 week ↑	
Weight gain during pregnancy (kg)	15 (11, 19)	16 (11, 19)	1.01 (0.97, 1.05)	0.32
	15 ± 6.2	15 ± 6.5	per 1 kg ↑	
Cesarean deliveries	198 (68.0 %)	53 (63.1 %)	0.83 (0.49, 1.41)	0.40
Birth weight (g)	3370 (3060, 3650)	4290 (4155, 4565)	–	<0.001
	3336 ± 397	4371 ± 306		

^*^Comparison of medians or frequencies in macrosomia vs. no macrosomia

Less than one half (41.6 %) of the women entered the intensive diabetes care program (pregnancy planning). Most patients during pregnancy were treated with rapid-acting insulin analogs (60.3 %) and predominantly used insulin pumps (58.9 %). HbA_1c_ decreased systematically during the gestational period: from median 6.4 % (46 mmol/mol) in the first trimester to 5.7 % (39 mmol/mol) and 5.6 % (38 mmol/mol) in the second and third trimesters, respectively.

**Fig. 2:**
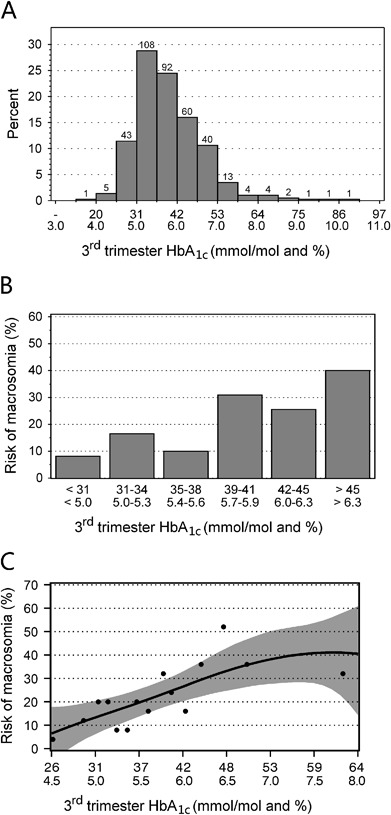
Third trimester HbA_1c_ and risk of macrosomia. **a** Distribution of 3rd trimester HbA_1c_. Numbers over the bars indicate number of women. **b** Risk of macrosomia in strata of 3rd trimester HbA_1c_. **c** Bayesian thin plate regression model illustrating relationship between the risk of macrosomia and 3rd trimester HbA_1c_. Dots indicate the mean HbA_1c_ and observed macrosomia risk in 15 strata containing 25 women each, black line indicates functional relationship between HbA_1c_ and macrosomia estimated from the observed data and gray area is 95 % credible interval around the fitted line

The proportion of women, who reached the therapeutic goal of HbA_1c_ < 6.1 % (43 mmol/mol) increased significantly from 36.5 % (137 women) in the 1st trimester to 67.2 % (252) and 71.5 % (268 women) in the 2nd and 3rd trimester, respectively. The distribution of HbA_1c_ in the 3rd trimester is shown in Fig. 2a. The trend of improving glycemic control was paralleled, to a lesser degree, by changes in mean daily glucose level indices from SMBG records in the 1st, 2nd, and 3rd trimester: median 5.9, 5.7, and 5.7 mmol/l, respectively, and by mean fasting glucose in SMBG records: 5.6, 5.2 and 5.1 mmol/l, respectively. The median weight gain of the women during pregnancy was 15 kg.

By design we analyzed term pregnancies; the median duration of gestation was 39 weeks. Most (66.9 %) pregnancies ended with cesarean delivery. The median birth weight was 3520 g. There were 84 (22.4 %) cases of macrosomia (>4000 g), a large proportion despite excellent glycemic control, especially in the 2nd and 3rd trimesters. However, more severe macrosomia was infrequent: there were 22 newborns (5.9 %) weighting >4500 g and only one with birth weight >5000 g. In comparison, among 70 cases of preterm deliveries (20th to 36th week, excluded from the further analysis) the median birth weight was 2900 g and there were only 5 cases of macrosomia.

In Table 1 we present comparison between woman–offspring pairs with and without macrosomia. Mothers of newborns with macrosomia were slightly younger (median 27 vs. 28 years, *p* = 0.026) and had longer (*p* = 0.029) duration of pregnancy, but did not differ from the rest in diabetes duration or BMI. Diabetic retinopathy at baseline had lower prevalence in mothers of macrosomic newborns (16.6 vs. 28.2 %). There were no differences in diabetes care: rates of pregnancy planning and analog insulin use were similar. Mothers of macrosomic newborns (with borderline significance: *p* = 0.059) were treated more frequently with personal insulin pumps (67.9 vs. 56.4 %). The most important difference between the macrosomia and non-macrosomia groups was observed for HbA_1c_, especially in the 2nd and 3rd trimesters: 6.0 % (42 mmol/mol) vs. 5.6 % (38 mmol/mol), *p* < 0.001 and 6.0 % (42 mmol/mol) vs. 5.5 % (37 mmol/mol ), *p* < 0.001, respectively. Similarly, we observed differences in the 2nd and 3rd trimester (but not the 1st trimester) in mean self-monitored glucose: 6.0 vs. 5.6 mmol/l (*p* < 0.001) and 5.9 vs. 5.7 mmol/l (*p* = 0.001), respectively. Similar differences were observed for mean postprandial glucose in self-monitoring, which was highly correlated with mean glucose. No significant differences were observed for mean glucose amplitude. There was also a significant difference across all three trimesters between mean fasting self-monitored glucose. In the 1^st^ trimester it was 6.1 vs. 5.5 mmol/l (*p* = 0.012), in the 2nd it was 5.7 vs. 5.1 mmol/l (*p* < 0.001), and in the 3rd trimester it was 5.4 vs. 5.0 mmol/l (*p* < 0.001).

In order to define a set of predictors that independently contribute to the risk of macrosomia in our cohort we used a logistic regression model. The resulting set of variables is shown in Table 2. Gestational age, within the examined range of 37 to 45 weeks, was strongly associated with birth weight. One additional week of pregnancy increased the odds of macrosomia 1.3-fold (*p* = 0.010). Maternal age (but not T1DM duration) was associated with lower risk of macrosomia (odds ratio 0.72 per 5 years increase, *p* = 0.027). The mother’s baseline (first visit) weight (but not BMI) was associated with a 1.13-fold increase in odds of macrosomia (per 5 kg increase), with borderline significance (*p* = 0.054), and this variable was retained in the final model for its possible confounding properties. The strongest predictor of birth weight was HbA_1c_ recorded in the 3^rd^ trimester. Its 1 percentage point increase was associated with a 1.68-fold increase in odds of macrosomia (*p* = 0.001). Independently from HbA_1c_, the level of mean fasting glucose (measured with personal glucose meter) in the 3rd trimester was associated with a 1.38-fold increase in odds of macrosomia (per 1 mmol/l increase, *p* = 0.005). There was no statistically independent association of mean blood glucose, mean glucose amplitude or mean postprandial glucose from SMBG records and risk of macrosomia. No marker of glycemia from the 1st or 2nd trimester had significant effect in the presence of the 3rd trimester markers.

**Table 2:** Predictors of macrosomia in multiple logistic model analysis and their association with birth weight in multiple regression model analysis

Predictor	Association with risk of macrosomia (logistic model)		Association with birth weight (linear regression model)	
	Odds ratio (95 % CI)	*P*-value	Estimate (95 % CI)	*P*-value
Duration of pregnancy (increase by 1 week)	1.27 (1.06, 1.52)	0.010	90 (53, 127)	<0.001
Mother’s age (increase by 5 years)	0.72 (0.53, 0.96)	0.027	−78 (−136, −21)	0.008
Mother’s baseline weight (increase by 5 kg)	1.13 (1.00, 1.27)	0.054	30 (4, 56)	0.022
3rd trimester HbA1c (increase by 1 %)	1.68 (1.22, 2.32)	0.001	136 (69, 204)	<0.001
3rd trimester mean fasting glucose (increase by 1 mmol/l)	1.38 (1.10, 1.72)	0.005	100 (54, 147)	<0.001

In linear regression effect estimates indicate change of predicted birth weight in grams

*CI* confidence interval

We also evaluated the predictors listed in Table 2 for their association with birth weight. The results are consistent with the findings from the logistic models. Briefly, in the model adjusted for maternal age, gestational age and the mother’s baseline weight, an increase of HbA_1c_ by 1 percentage point was associated with a 136 g higher birth weight (*p* < 0.001), and in addition, a 1 mmol/l increase in mean fasting glucose (SMBG) was associated with a 100 g increase in birth weight (*p* < 0.001), independently from HbA_1c_.

As a sensitivity analysis, we restricted our study group to women whose HbA_1c_ was <6.5 % (48 mmol/mol) in all three trimesters. There were 184 such women, and the risk of macrosomia in this subgroup was 16.3 %. Third trimester HbA_1c_ remained a significant predictor of macrosomia (odds ratio 2.87, 95 % CI: 1.17, 7.03, *p* = 0.022) when adjusted for maternal age, gestational age and the mother’s baseline weight. It remained significantly associated with birth weight (247 g increase per 1 % HbA_1c_ increase, 95 % CI: 95, 399; *p* = 0.002). In 124 women with HbA_1c_ < 6.1 % (43 mmol/mol) there were too few cases of macrosomia (*n* = 17) to fit a multiple logistic model, but birth weight remained dependent from 3rd trimester HbA_1c_ (259 g increase per 1 % HbA_1c_ increase, 95 % CI: 55, 464; *p* = 0.013).

To further dissect the relationship between the birth weight and HbA_1c_ in the 3rd trimester, for illustrative purpose, we estimated macrosomia risk in six arbitrary strata of HbA_1c_, as shown in Fig. 2b. Although there was an apparent increase in macrosomia risk in women with HbA_1c_ >5.6 % (38 mmol/mol), there was no statistically significant difference in association between macrosomia and HbA_1c_ below and above this threshold (*p* = 0.15). To formally determine the shape of the relationship between HbA_1c_ in the 3rd trimester and macrosomia and establish a possible target value of this marker we estimated the risk of this complication in 15 strata containing 25 women each, ranked by HbA_1c_. We plotted the risks against mean HbA_1c_ level in each stratum (black dots in Fig. 2c) and fitted a Bayesian penalized splines model to this scatter. It provided a continuous, nonlinear functional description of the relationship between HbA_1c_ and macrosomia risk with 95 % Bayesian credible interval (black line and gray area, respectively in Fig. 2c). Inspection of this relationship reveals that it is linear in the HbA_1c_ range from 4.5–7.0 % (26 to 53 mmol/mol). In this HbA_1c_ range, the Bayesian estimate of macrosomia risk increase per 1 % increase in HbA_1c_ was 12.7 percentage points. The risk of macrosomia predicted from our data (Fig. 2c) for the 3rd trimester HbA_1c_ 6.0 % (42 mmol/mol) was approximately 25 %. The risk was 10 % for HbA_1c_ level < 5.0 % (31 mmol/mol).

## Discussion

This large observational one-center study has demonstrated high (above 20 %) prevalence of macrosomia in T1DM-complicated pregnancies. This phenomenon occurred in spite of achieving the HbA_1c_ goal of <6.1 % in the 2nd and 3rd trimester by the majority of women.

A quarter century ago, the St Vincent’s Declaration established, among many others, the goal of improving outcomes of pregnancies complicated by diabetes to make them comparable with those observed in non-diabetic women [22]. In spite of this, as proven in many clinical observations, macrosomia (defined either as birth weight above 4000 g or above the 90th percentile) is still very common in T1DM pregnancies with prevalence of 20–50 % [5, 8, 23]. Moreover, there has been very little evidence for the reduction of this most common neonatal outcome of T1DM-complicated pregnancy. There were reports from several European countries showing no significant decrease between surveys performed at different time points over the last two decades [24–26]. A rare example of an improvement was reported from Ireland, where the Irish Atlantic Diabetes in Pregnancy (ATLANTIC DIP) group representing five antenatal centers coordinated specialized care for women with diabetes before, during, and after pregnancy [27]. This group reported a 30 % drop from the initial number of neonates born with macrosomia in women with T1DM [28]. However, their initial HbA_1c_ level was substantially higher than in our cohort.

Our finding not only confirms but also broadens and deepens the conclusions of a study [23] performed in the population of Northern England (Northern Diabetes in Pregnancy Survey, NORDIP), where the risk of large for gestational age (LGA) birth weight increased steeply and monotonically with HbA_1c_ in the range of 5.5–7.0 % (37–53 mmol/mol). In our study group the median HbA_1c_ levels in the 2nd and 3rd trimester were 1 % lower than it was recorded in the NORDIP study. Thus, we can extend this observation towards the lower tail of HbA_1c_ distribution. To avoid possible impact of arbitrary stratification of HbA_1c_ distribution and random variation, we used a penalized thin-plate spline model to obtain a flexible functional representation of the relationship between the 3rd trimester HbA_1c_ and macrosomia. Such function can adapt to possible non-linear relationships, thresholds and plateaus in analyzed data. We were unable to identify any risk threshold in HbA_1c_ distribution. There was a linear relationship between the 3rd trimester HbA_1c_ and the risk of macrosomia, starting from values of this marker as low as 4.5 % (26 mmol/mol). In our patients the risk of macrosomia was elevated (more than 10 %) for HbA_1c_ >5.0 % (31 mmol/mol). Interestingly, as in the NORDIP study, the risk levelled for HbA_1c_ values >7.0 % (53 mmol/mol). The risk levels, however, cannot be compared directly between that study and ours, because we used macrosomia, rather than percentile chart-based LGA and SGA (small for gestational age) births.

In a recently published analysis of the relationship between pregnancy complications and HbA_1c_ in the Diabetes and Pre-eclampsia Intervention Trial (DAPIT) population the risks of LGA births in strata of 2nd and 3rd trimester HbA_1c_ were strikingly similar to the ones observed in the NORDIP study [29]. There was a similar linear increase of risk followed by a plateau for HbA_1c_ values >7.0 % (53 mmol/mol). To the best of our knowledge NORDIP and DAPIT are the only cohorts in which linearity of the association between LGA and HbA_1c_ in pregestational diabetes has been formally explored. The unique value of our study is that it includes a large number (184) of women with HbA_1c_ during all pregnancy below the current widely used therapeutic target of 6.5 %. Our results strongly suggest that macrosomia risk remains glucose-dependent for HbA_1c_ levels that are significantly lower. Our finding can be also corroborated by the results of the HAPO study, which has shown that macrosomia risk can be increased in women with OGTT glucose values previously perceived as normal.

The strongest and the most consistent predictor of macrosomia is 3rd trimester HbA_1c_. In the SMBG profile association independent from HbA_1c_ was observed only for mean fasting glucose. Neither mean glucose amplitude nor postprandial SMBG glucose were associated with macrosomia, and mean glucose was a much weaker predictor than HbA_1c_. This likely reflects deficiency of glucometer measurements in capturing all the variability of glucose levels. Another possible—although probably less likely—explanation could be the self-reporting nature of analyzed measurements and preferential inclusion of in-target glucose values by the patients.

The main finding of our study prompts urgent need for further research into effective means of glucose monitoring in pregnancy and contradicts the recent shift of target HbA_1c_ in pregnancy from <6.0 % (42 mmol/mol) to <6.5 % (47 mmol/mol) by NICE and ADA [16, 17]. Despite improvements in diabetes management, macrosomia rates in T1DM are consistently reported as at least two-fold higher than in the general population. Attaining currently recommended HbA_1c_ level does not prevent LGA births and, as a therapeutic target, it is misleading. HbA_1c_ values >4.5 % (26 mmol/mol) remain associated with macrosomia risk, however, decreasing HbA_1c_ to <6.0 % (42 mmol/mol), values comparable with observed in the non-diabetic general population [30] is not feasible in clinical practice. There is an urgent need to improve the standards of glucose monitoring during pregnancy, possibly with wider use of continuous glucose monitoring. Alternative markers of hyperglycemia, such as 1,5-anhydroglucitol, which, according to our earlier finding [31], was a better marker of maternal glycemic control and predictor of neonatal birth weight than HbA_1c_ in pregnancies complicated by T1DM, may possibly find wider application.

This study has several potential shortcomings. It is just observational research, meaning it is prone to some typical biases, for example some missing data or unprecise self-reported glucose measurements. Such biases could possibly alter the study results, for example by underestimating the association between the mean glucose amplitude and the risk of macrosomia. We believe that our study is representative for the T1DM population of the Lesser Poland region, however, we could not exclude that some women remained under medical care elsewhere and did not participate in this study. This is, however, unlikely as medical care of pregnant T1DM women in Poland is centralized and it is strongly recommended to refer the patients during pregnancy planning or at the latest in early pregnancy to tertiary reference centers like the Department of Metabolic Diseases in Krakow. Additionally, two limitations related to laboratory diagnostics should be acknowledged. First, there were different types of glucose meters used by the patients from our study over the period of 15 years. Nevertheless, these were devices from leading global manufacturers, all of them meeting European accuracy standards. We also did not control factors potentially affecting the HbA_1c_ level during complicated pregnancy, such as iron or folic acid deficiency anemia. While we cannot entirely exclude the influence of these factors on the study results, this seems to be unlikely.

There are two more important issues that should be discussed. We chose to use as the outcome macrosomia defined as birth weight above 4000 g rather than LGA (large for gestational age) weight. This was related to several factors. First, we are lacking methodologically correct percentile charts from the Polish population for the study period. Second, there are scientific arguments for such a choice, since the 4 kg cut-off is a clinically important indicator of potential complications [8, 23, 26]. Additionally, possible injuries in the newborn or to the birth canal depend more on the absolute rather than relative size of a fetus. Moreover, although LGA is widely used in many countries, its definition relies on the quality and accuracy of percentile charts, which may differ between particular regions or time periods. A 4 kg cutoff is easy to compare between different studies. Finally, we study only term pregnancies and, additionally, adjust the risk of macrosomia in the regression models to gestational age. The second issue is related to the fact that both maternal weight and BMI were used as candidate independent variables. We assumed that birth weight may depend not only on mother’s body fat, but possibly also on her overall body size. Our analysis showed that maternal pregestational weight, unlike BMI, was indeed associated with birth weight.

In summary, despite an in-target HbA_1c_ level in most women, the risk of macrosomia remains high in pregestational T1DM, and it remains a glucose-dependent complication. Our study suggests inadequacy of HbA_1c_ and self-monitoring, as they apparently miss significant episodes of hyperglycemia.
